# Live Birth Rates after Active Immunization with Partner Lymphocytes

**DOI:** 10.3390/biomedicines9101350

**Published:** 2021-09-29

**Authors:** Veronika Günther, Ibrahim Alkatout, Lisa Meyerholz, Nicolai Maass, Siegfried Görg, Sören von Otte, Malte Ziemann

**Affiliations:** 1 Department of Obstetrics and Gynecology, University Hospitals Schleswig-Holstein, Campus Kiel, Arnold-Heller-Straße 3 (House C), 24105 Kiel, Germany; ibrahim.alkatout@uksh.de (I.A.); lisa.meyerholz@web.de (L.M.); Nicolai.maass@uksh.de (N.M.); 2 University Fertility Center, Ambulanzzentrum gGmbH, University Hospitals Schleswig-Holstein, Campus Kiel, Arnold-Heller-Straße 3 (House C), 24105 Kiel, Germany; soeren.vonotte@uksh.de; 3 Institute for Transfusion Medicine and Transplant Center, University Hospitals Schleswig-Holstein, Campus Kiel, Arnold-Heller-Straße 3 (House 17), 24105 Kiel, Germany; siegfried.goerg@uksh.de (S.G.); malte.ziemann@uksh.de (M.Z.); 4 Institute for Transfusion Medicine and Transplant Center, University Hospitals Schleswig-Holstein, Campus Lübeck, Ratzeburger Allee 160 (House 31), 23538 Lübeck, Germany

**Keywords:** recurrent miscarriage, implantation failure, immunization, paternal lymphocytes, live birth rate

## Abstract

Although many potential causes have been established for recurrent implantation failure (RIF) and recurrent miscarriage (RM), about 50% of these remain idiopathic. Scientific research is focused on immunological risk factors. In the present study, we aim to evaluate live birth rates after immunization with paternal lymphocytes (lymphocyte immunotherapy (LIT)). This retrospective study consisted of 148 couples with a history of RM and/or RIF. The women underwent immunization with lymphocytes of their respective partners from November 2017 to August 2019. Fifty-five patients (43%) had live births. Stratified by indication (RM, RIF, combined), live birth rates in the RM and the combined group were significantly higher than that in the RIF group (53%, 59% and 33%, respectively, *p* = 0.02). The difference was especially noticeable during the first 90 days after immunization (conception rate leading to live births: 31%, 23% and 8% for RM, the combined group and RIF, respectively; *p* = 0.005), while there was no difference between groups during the later follow-up. LIT was associated with high live birth rates, especially in women with recurrent miscarriage. In view of the limited data from randomized studies, LIT cannot be recommended as routine therapy. However, it may be considered in individual cases.

## 1. Introduction

Recurrent miscarriage (RM) affects 1–2% of couples who attempt to conceive and has been defined as three consecutive pregnancy losses prior to 20 weeks of gestation from the last menstrual period [1,2]. According to the American Society for Reproductive Medicine (ASRM), RM may be diagnosed in women after two or more pregnancy losses with clinical (ultrasonography or histopathology) evidence of pregnancy [3]. Recurrent pregnancy loss is a common reason for seeking medical help in a fertility clinic, as couples like to rule out the likely reason for abortions. Apart from couples with RM, those with recurrent implantation failure (RIF) after assisted reproductive technology (ART) also seek advice in order to establish the cause of the disorder and its treatment [4,5,6]. Potential causes include parental chromosomal anomalies, genetic or metabolic abnormalities of the embryo, hypothyroidism, insulin resistance, coagulation disorders, autoimmune diseases, endometriosis, anatomic uterine abnormalities, or chronic endometritis [7]. Celiac disease is an autoimmune disorder of the small intestine associated with several extra-intestinal features, such as reproductive disorders. Undiagnosed celiac disease is a risk factor for infertility [8,9]. The pathomechanism responsible for reproductive complications is not completely understood. Nevertheless, women with a history of RM or RIF should be screened for celiac disease. Adoption of a gluten-free diet could have a positive impact on fertility in these patients [10].

In view of the large number of potential reasons for infertility, even an extensive diagnostic investigation may not reveal the specific reason.

As approximately 50% of cases are idiopathic, research has been focused on immunological risk factors.

For the female immune system, the embryo represents a semi-allogeneic transplant because half of the embryo’s genes are of paternal origin. Instead of a conventional immune response, the embryo induces a secondary protection mechanism [11]. From the development of the blastocyst through to implantation, an intensive immunological interaction is required between the embryo and the maternal immune system. Trophoblast invasion into the maternal decidua is influenced by immunological effector cells, particularly uterine natural killer cells (uNK). With the help of the vascular endothelial growth factor (VEGF) and interferon gamma (INF-gamma), they stimulate the conversion of the spiral arteries and are involved in regulating the depth of invasion [12,13].

Immunological treatment options include intralipids, corticosteroids, intravenous immunoglobulins, tumor necrosis factor (TNF)-alpha-blocker, granulocyte-colony-stimulating factor (G-CSF), or immunization with partner or third-party lymphocytes [14]. The aim of lymphocyte immunotherapy is to establish anti-paternal cytotoxic antibodies, anti-idiotypic antibodies (Ab2) and mixed lymphocyte reaction blocking antibodies (MLR-Bf), reduced NK cell activity, improved Th-1/Th-2 balance with Th-2 predominance, and an improved regulatory T (Treg) cell profile in order to create a favorable immune environment for embryo implantation [15,16,17].

Whether the above-mentioned antibodies themselves promote implantation or whether the antibodies serve as surrogate markers has not been established yet.

Since November 2017, we have been treating couples with recurrent miscarriage and/or recurrent implantation failure at the Institute of Transfusion Medicine, University Clinic of Schleswig-Holstein (UKSH), Campus Kiel, with active immunization by intracutaneous application of the partner’s lymphocytes. In the following, we analyze the outcomes after immunization with paternal lymphocytes in patients with RM and/or RIF.

## 2. Material and Methods

Lymphocyte immunotherapy (LIT) was initially established in the 1980s and performed until 2014 at the Institute of Immunology, UKSH, Campus Kiel. Special requirements were then imposed by the drug regulatory authorities, which made it necessary to restructure the manufacturing process. The treatment was discontinued and then resumed at the end of 2017.

### 2.1. Patients

One hundred and forty-eight consecutive patients undergoing immunization with partner lymphocytes from November 2017 to August 2019 were analyzed retrospectively. Data were collected from August to November 2020. Thus, at least one year elapsed between immunization and the evaluation of outcomes. The evaluation was conducted by telephone. The ethics committee of the Medical Faculty of the Christian Albrechts University of Kiel (Arnold-Heller-Str., House no. 9, 24105 Kiel, Germany) approved the study (Vote no. B 555/20). Informed consent was obtained from all participants in the study of 148 patients, and 129 of these could be interviewed on the phone and were included in the analysis.

Based on their history, the patients were divided into three groups:

1. Patients with recurrent miscarriage only (32);
2. Patients with implantation failure only (75);
3. Patients with a combination of both (22).

The primary endpoint was the live birth rate after immunization in each of the three groups. In addition, the mode of delivery (cesarean section vs. spontaneous birth), the time interval between immunization and conception leading to live birth, and between immunization and birth were investigated. A potential dose dependence was also examined.

Couples with RIF after ART and/or those with RM were referred from their fertility clinics to our outpatient department at the Institute of Transfusion Medicine. One hundred and twenty of 129 couples were German. The remaining couples were from Austria, Switzerland, Luxembourg, Hungary, Poland, and the United Arab Emirates.

### 2.2. Indications for LIT

The patients had to fulfill the following criteria in order to be approved for immunization: at least two clinical pregnancies that culminated in an abortion before the 24th week of gestation (RM) or at least two cycles of in vitro fertilization or intracytoplasmic sperm injection with at least two embryos of good quality in each transfer without pregnancy (implantation failure), unremarkable/normal results for coagulation tests, tests for autoimmune diseases (antiphospholipid antibodies, antinuclear antibodies, thyroid antibodies), hysteroscopy, a glucose tolerance test, and karyotyping of both partners. A body mass index of 30 (kg/m^2^) was the upper weight limit for women. The age limit for women was 45 years.

### 2.3. Contraindications for LIT

Couples were excluded from immunization if they had had an abortion after 24 weeks of gestation or a live birth with the same partner in the past without prior immunization.

Women who suffered from a systemic autoimmune disease, such as lupus erythematosus, antiphospholipid syndrome, Crohn’s disease, ulcerative colitis or multiple sclerosis, chronic diseases that may necessitate a transplant at a later date (diabetes mellitus, cystic fibrosis, polycystic kidney disease) or transplants in their medical history were excluded from immunization. If the partner was subject to a high risk of passing on an infectious disease or malignant cells, he was not accepted for a lymphocyte donation.

### 2.4. Human Leukocyte Antigen (HLA) and Human Platelet Antigen (HPA)

Typing for HLA-A and -B of both partners was performed before immunization. Typing of HLA-C and/or class II alleles was applied to couples who had identical A and B antigens. All HLA typing was performed by sequence-specific oligonucleotide probe molecular typing (LABType, OneLambda, Carnoga Park, CA, USA). If the partner only possessed HLA alleles and those HLA alleles were found equally in the patient’s cells, the couple were excluded from treatment. HLA antibodies against the partner’s lymphocytes were measured before and one month after LIT with a Luminex PRA test (LABScreen PRA, OneLambda, Carnoga Park, CA, USA).

Additionally, HPA-1 was determined before immunization (Fluogene HPA1a/b, InnoTrain, Dreieich, Germany). Human platelet antigens are polymorphisms in platelet antigens and can stimulate the production of alloantibodies in recipients of transfused platelets from donors with different HPAs. These antibodies may cause fetal and neonatal alloimmune thrombocytopenia (FNAIT) with severe bleeding and the risk of perinatal death or lifelong disability. In cases of suspected HPA antibody development, the couple were excluded from immunization.

### 2.5. Lymphocyte Immunotherapy

**Figure 1:**
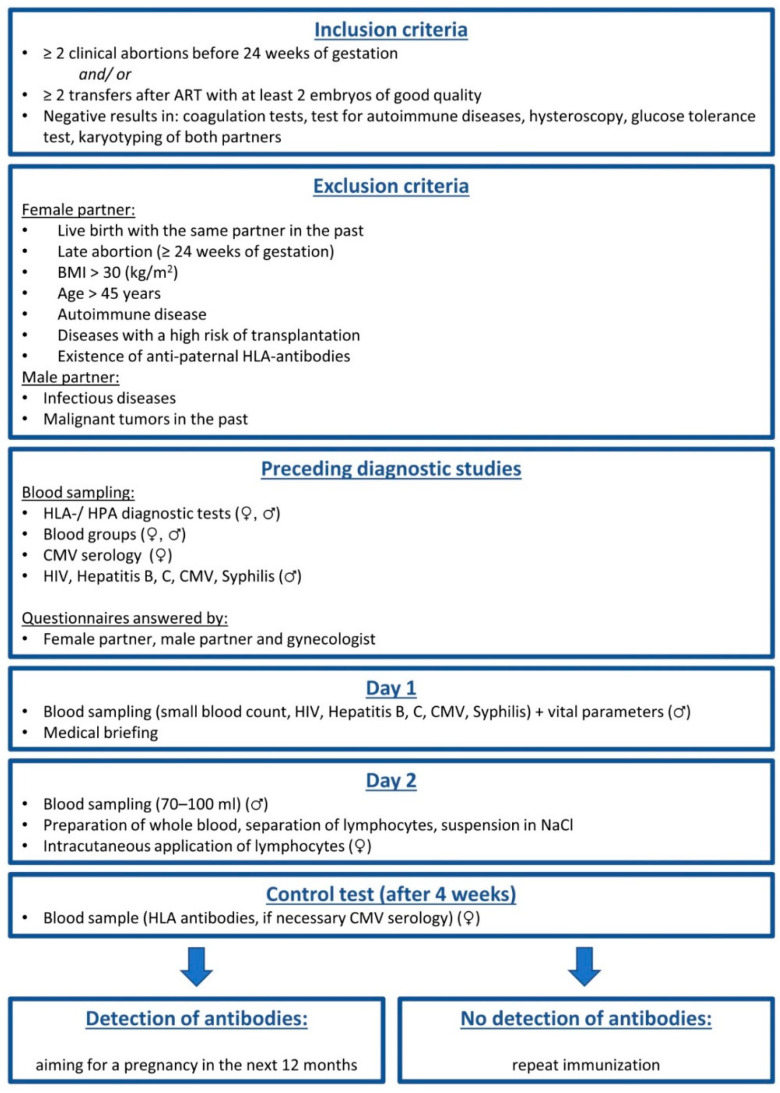
Schematic sequence of immunization. Abbreviations: ART: assisted reproductive technology, HLA: human leukocyte antigen, HPA: human platelet antigen, CMV: cytomegalovirus.

Usually 70 mL (100 mL in cases of a lymphocyte count below 2 × 10^6^/ mL) of heparinized blood was taken from the male partner. The lymphocytes were separated under sterile conditions by Ficoll-Hypaque density gradient centrifugation, and up to 70 × 10^6^ lymphocytes were re-suspended in 1 mL of normal saline. The suspension was given to the female partner by 10–15 intradermal injections on the volar aspect of a forearm. The suspension was not stored but applied 4–5 h after blood withdrawal. In cases of a Rhesus-negative patient and a Rhesus-positive partner, we performed anti-D prophylaxis with Rhophylac 300^®^ (CSL Behring, Marburg, Germany). Four weeks later we tested for anti-paternal HLA antibodies and recommended a further LIT if the test was negative. Figure 1 shows the sequence of immunization.

### 2.6. Statistical Analysis

Quantitative values are presented as the median (range). Differences between groups were evaluated by two-sided chi-square-tests or Kruskal–Wallis tests as appropriate. Alpha adjustment for multiple testing was not performed, and the results were interpreted accordingly. SPSS Statistics 25 (SPSS Inc. an IBM Company, Chicago, IL, USA) was used for statistical calculations.

## 3. Results

A total of 129 women had complete follow-up data (87%) and were included in this retrospective study. Thirty-two patients suffered from recurrent miscarriage, 75 had implantation failure, and 22 had a combination of both.

There was no significant difference in female age, male age, interval between LIT and conception leading to live birth, delivery after immunization, and follow-up period between the three groups (RM, RIF, and combined). Except for the mode of delivery, all p-values were *p* ≥ 0.1. Table 1 shows patient characteristics in detail. Fifteen women were older than 39 years (3 with RM, 7 with RIF, and 5 in the combined group).

Seventeen patients (53%) with RM, 25 (33%) with RIF and 13 (59%) in the combined group had a live birth after immunization. In total, 55 patients (43%) had a live birth after immunization.

**Figure 2:**
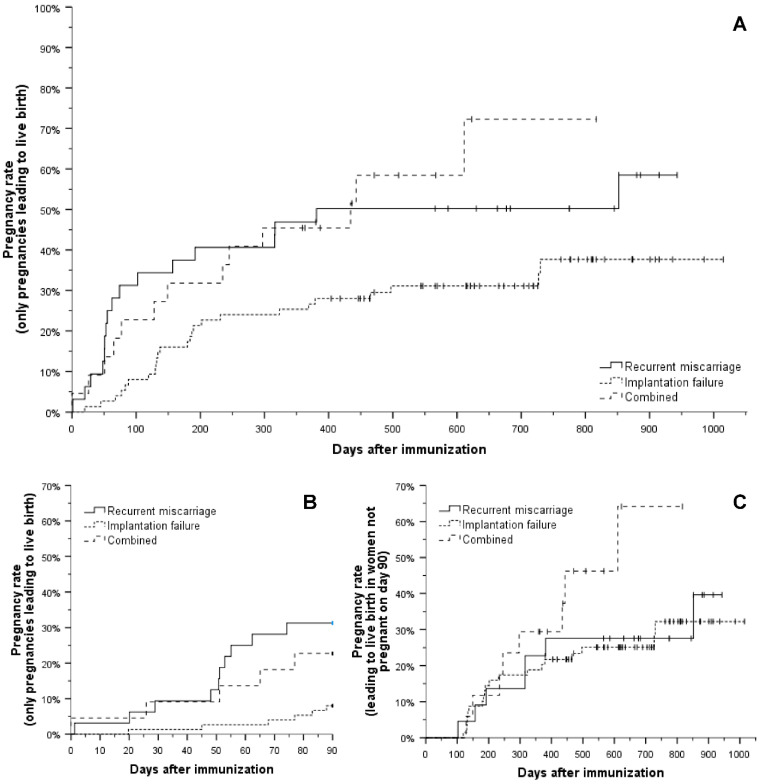
Conception rate after immunization resulting in a live birth. (**A**): The pregnancy rate leading to live birth was lower in patients with implantation failure only (overall *p* = 0.02, *p* = 0.03 compared to patients with recurrent miscarriage, *p* = 0.008 compared to patients with combined recurrent miscarriage and implantation failure), but not different between patients with recurrent miscarriage and those with combined recurrent miscarriage and implantation failure (*p* = 0.65). (**B**): Pregnancy rates differred mainly during the first 90 days after immunization, with lower rates in patients with implantation failure only (overall *p* = 0.005; *p* = 0.001 compared to patients with recurrent miscarriage, *p* = 0.04 compared to patients with combined recurrent miscarriage and implantation failure), but no significant difference was observed between patients with recurrent miscarriage and patients with combined recurrent miscarriage and implantation failure (*p* = 0.49). (**C**): There was no significant difference in pregnancy rates leading to live birth after day 90 post immunization (overall *p* = 0.18).

Live birth rates were significantly lower in patients with RIF compared to patients with RM or the combined group, while the difference between the latter two groups was not significant (Figure 2A). Interestingly, this difference was only detectable during the first 90 days after immunization (Figure 2B), but not during the subsequent follow-up (Figure 2C). Thirty-one percent of patients with RM and 23% of patients in the combined group conceived (and eventually had live births) during the first 90 days after LIT, compared to 8% of women with RIF.

Thirty-one patients (60%) gave birth vaginally while 21 patients (40%) had a cesarean section. The data are shown in Table 1. The two-sided chi-square test concerning the mode of delivery was significant (*p* < 0.05). However, this outcome was most likely a coincidental result of multiple testing.

Table 2 shows the height and birth weight of the newborns. No malformations or diseases were observed in any child.

No significant differences in live birth rates were registered in relation to the number of injected lymphocytes (data not shown). Lymphocyte counts of 30–45 × 10^6^ /mL were applied most frequently. Likewise, repeat immunization due to undetected or weakly detected anti-paternal antibodies after the first treatment was not associated with different live birth rates.

## 4. Discussion

Forty-three percent of patients gave birth to a live infant after LIT. Live birth rates differed significantly according to the indication for LIT, and were significantly higher in women with recurrent miscarriage or RM combined with RIF compared to RIF only (53% and 59% vs. 33%). Especially, conception rates (leading to live births) during the first 90 days after immunization were significantly higher in cases of recurrent miscarriage compared to RIF (32% vs. 8%, *p* = 0.001).

A limitation of the present study was the absence of a control group. Instead, we compared the RIF group with published data from fertility clinics at which live birth rates were studied in relation to the number of previous ART cycles. Previous studies on LIT employed control groups that did not receive LIT. We used these groups as controls for our patients who were given LIT.

Table 3 summarizes studies addressing live birth rates in patients with RIF in relation to the number of previous ART cycles who did not undergo LIT [18,19,20]. Although the live birth rates reported by Koot et al. were significantly higher than those registered in our study (49% vs. 33%) [19], a comparison of these two analyses is limited by the following factors. First, 7 of 75 patients with RIF in our study (9%) were older than 39 years. Second, the follow-up period of 66 months in the former study was significantly longer than our follow-up of 11.8 to 34.2 months.

Leijdekkers et al. analyzed cumulative live birth rates over multiple complete ART cycles over 18 months of treatment [20]. All live births, irrespective of the mode of conception, were taken into account. The cumulative birth rate of 56% reported by the authors is higher than that registered in our study, but the comparability of these data is hindered by the fact that Leijdekkers et al. included patients receiving their first ART cycle as well [20]. Our study consisted solely of patients who had undergone at least two unsuccessful ART cycles. Leijdekkers et al. also analyzed live birth rates in relation to the number of ART cycles, and observed a continuous fall in live birth rates after consecutive ART cycles (Table 3).

Compared to the other cited studies, we registered higher live birth rates after immunization in women with RIF than in control groups derived from the published literature who did not undergo LIT.

With regard to RM patients, Table 4 lists selected studies that address the effect of LIT compared with controls who did not receive treatment. Study protocols varied considerably with respect to the mode of application and the timing of immunization (before and/or during pregnancy). Although our live birth rates after LIT in couples with RM were lower than those of the treatment groups in the cited studies, our success rate after LIT was higher than those reported for control groups in the studies, and approximately the same as the figures reported by Yanping et al. [21,22,23,24].

An interesting aspect of our study was the high conception rate leading to live births, especially during the first 90 days after immunization. We do not know whether this effect was due to the LIT itself and its impact on immunomodulation or whether it was a psychological effect of the treatment.

Couples with RIF or RM are prone to heightened anger, depression, anxiety, and feelings of grief and guilt [25]. These psychological aspects must be given special attention throughout follow-up evaluations and during ensuing pregnancies. Stray-Pedersen et al. analyzed the influence of specific psychological support known as tender loving care (TLC) in couples with recurrent miscarriage [26,27]. A cohort of 158 couples with ≥ 3 consecutive pregnancy losses and no identifiable etiology were divided into two groups: one received routine obstetric care during the next pregnancy (*n* = 42), while the other received additional tender loving care (*n* = 116). TLC was defined as psychological support with weekly medical and ultrasound examinations, instructions to avoid heavy work, travel and sexual activity. The difference in live births was significant: 36% in the control group and 85% in the TLC group. Despite these results, the study should be interpreted with caution. The groups were not randomized. The only inclusion criterion for the TLC group was the practicability, namely, the distance between the residence and the hospital.

Clifford et al. analyzed this aspect and reported that supportive care in early pregnancy conferred a significant beneficial effect on pregnancy outcomes. A miscarriage rate of 26% was noted in couples with close ties to the clinic, compared to 51% who did not participate in the TLC program [1,28]. Despite these notable findings, TLC is neither the routine standard of care in Germany nor the focus of current studies.

Randomized controlled trials (RCTs) are essential for any differentiation between the direct effect of LIT and a likely placebo effect. Two recent meta-analyses of RCTs pointed to higher live birth rates in patients with idiopathic RM who received LIT. Cavalcante et al. performed a systematic review and meta-analysis of published data about the efficacy and safety of immunization with paternal lymphocytes in cases of RM [17]. Six published meta-analyses were retrieved; two reported no improvement in live birth rates after the use of immunization, and four registered a beneficial effect of immunotherapy with lymphocytes in cases of RM, with significant improvements in live birth rates. The two most relevant meta-analyses (Cochrane and Liu et al.) and recent data reported by Cavalcante et al. revealed a positive impact of LIT (OR 3.22, 95% confidence interval (CI) 2.74–3.78, *p* < 0.00001) [24,29,30]. The authors concluded that immunotherapy is an efficient and safe procedure in cases of recurrent miscarriages with no identifiable cause [17].

The Cochrane analysis of 2014 differed from the above-mentioned data. Wong et al. included 12 randomized controlled trials comparing paternal white cell immunization versus placebo, comprising 641 women with recurrent miscarriages in the past. Women who were treated with paternal cell immunization were not at increased odds for live births compared to those given placebo, with an OR of 1.23 and a confidence interval (CI) of 0.89–1.69. The intention-to-treat analysis yielded no significant differences between paternal cell immunization treatment and controls in terms of subsequent live births (four trials, 350 women) OR 1.38, 95% CI 0.90–2.1 [30].

The results of this Cochrane analysis were criticized by a number of scientists [31,32]. The main point of criticism was the inclusion of the study by Ober et al. [33], who used different conditions for their preparation of lymphocytes. This was the only investigation that yielded a negative effect after LIT and an increase in miscarriage rates.

Ober et al. stored the partner’s blood (from which the lymphocytes were to be prepared) overnight at a temperature of 1–6 °C in order to extend the time period from blood withdrawal to immunization. Clark et al. showed that sufficient numbers of CD200+ cells are needed to achieve an immunomodulatory effect in immunotherapy with lymphocytes. CD200 is expressed on dendritic cells, among others, and can induce immunomodulation in the recipient in the course of immunization. Storage at low temperatures and for several hours reduces the CD200+ cell count [34,35].

Furthermore, Ober et al. included patients with autoimmune diseases, which may have had a negative effect on the results of immunotherapy with lymphocytes [33]. Further points of criticism were the lack of success monitoring (detection of anti-paternal HLA antibodies) following immunization, different methods used for the application of lymphocytes (intradermal, subcutaneous, and intravenous), as well as different dosages and lymphocyte concentrations [31,32,35].

A repeat analysis of the data from the Cochrane library, excluding the results of Ober et al., yielded a significant increase in live birth rates after immunization with partner lymphocytes (OR 1.63, 95% CI 1.13–2.35; *p*  =  0.009) [30].

A fundamental problem of the individual studies included in the meta-analyses mentioned above is their limited comparability due to different study protocols. The modes of application were intracutaneous, subcutaneous, or intravenous. Immunization was performed exclusively before pregnancy in some studies, and during pregnancy as well in others [29,30].

LIT has been used since the early 1980s. At the time, ART was still in a very early stage of development and was not part of clinical routines [36]. Therefore, the initial studies dealt with RM and not RIF. We still lack RCTs analyzing the effect of LIT in cases of RIF. Cavalcante et al. performed an update of a recent meta-analysis concerning immunotherapy and included the question of efficacy in RIF. No significant improvement in live birth rates was observed in couples with RIF [37].

Carp et al. confirmed these findings, and emphasized the fundamental difference in immunological mechanisms underlying implantation failure and recurrent miscarriage [38]. Nevertheless, the exact mechanism underlying the effect of LIT on the immune system in RIF and RM patients remains unclear [14,39].

The present study revealed higher live birth rates in RIF patients compared with controls. However, live birth rates were higher in cases of RM than RIF. This supports the hypothesis of different immunological mechanisms being responsible for implantation failure and recurrent miscarriage.

International guidelines are very cautious about recommending LIT and mention possible complications that could be caused by immunization, such as transmission of infections, formation of irregular antibodies, or autoimmune disorders [40,41,42].

Our treatment protocol included strict contraindications for preventing the above mentioned complications. The patients’ partners were tested for infectious diseases for several weeks prior to, as well as the day before immunization. A questionnaire was used to identify and exclude risk groups for the transmission of infection. Patients with autoimmune diseases were excluded from immunization in order to prevent aggravation of the disease. Furthermore, HPA1 typing was performed in both partners to identify risk constellations for the production of alloantibodies that may cause fetal and neonatal alloimmune thrombocytopenia (FNAIT). In cases of a potential risk of HPA antibody development, the couple were excluded from immunization.

## 5. Conclusions

The results of the present study suggest that immunization with paternal lymphocytes is associated with a positive effect on live birth rates. A high conception rate, leading to live births, during the first 90 days after immunization was noted especially in women with recurrent miscarriage. In view of the fact that even supportive therapies have yielded a beneficial effect in other studies and the body of insufficient data from randomized studies on LIT, our data do not justify recommending LIT as a routine therapy. However, after a careful review of indications and contraindications, immunization with partner lymphocytes may be discussed with the couple on an individual basis. It may be used as an ultima ratio, provided that all other potential causes of RM or RIF have been ruled out in advance.

## Figures and Tables

**Table 1:** Patient characteristics.

	Recurrent Miscarriage	Implantation Failure	Combination of Both	Total
Female age at immunization	35 (29–40)	35 (27–45)	37 (29–44)	36 (27–45)
Male age at immunization	37 (23–56)	38 (28–54)	39 (29–48)	38 (23–56)
Interval between LIT and conception leading to live birth (months)	1.9 (0–12.5)	4.5 (0.7–24)	4.9 (0–20.1)	4.3 (0–24)
Delivery (months after immunization)	11.1 (8.3–21.8)	13.8 (9.6–33.7)	13.7 (9.1–28.6)	13.2 (8.3–33.7)
Vaginal delivery (%)	69	70	31	60
Caesarean section (%)	31	30	69	40
Follow-up (months)	27.7 (12.5–34)	24.7 (13.3–34.2)	25.1 (11.8–34.0)	25.5 (11.8–34.2)

Data given as median values (range) or percentages, LIT: lymphocyte immunotherapy.

**Table 2:** Newborn characteristics.

	Female	Male	Total
Height (cm)	51 (38–59)	51 (38–58)	51 (38–59)
Weight (g)	3350 (1265–4284)	3380 (1260–4200)	3365 (1260–4284)
Weeks of gestation (months)	9.0 (6.8–9.5)	9.0 (6.8–9.6)	9.0 (6.8–9.6)

Data are given as median values (range).

**Table 3:** Summary of selected studies on live birth rates in RIF patients without additional treatment compared to live birth rates after LIT in our study.

Author	Year	Age, Median (Range)	Number of Patients	Observation Period	Follow-Up Period (Months), Median (Range)	Comment
Smith et al.	2015	35 (18–55)	156.947	2003–2010	n.a.	Live birth rates in relation to the number of ART cycles After the 1st cycle: 32% After the 2nd cycle: 27% After the 3rd cycle: 24% After the 4th cycle: 21% After the 5th cycle: 19% After the 6th cycle: 17%
Koot et al.	2019	n.s. (n.s.–39) *	118	2008–2012	max. 66	Cumulative live birth rate: 49% (95% CI 39–59%), calculated median time to pregnancy leading to live birth: 9 months.
Leijdekkers et al.	2019	34.4 (n.s.–44)	551	2011–2014	18	Cumulative live birth rate after 18 months: 56%. Live birth rates in relation to the number of ART cycles After the 1st cycle: 28% After the 2nd cycle: 27% After the 3rd cycle: 25% After the 4th cycle: 12% After the 5th + 6th cycle: 0%
Günther et al.	2021	35 (27–45)	129	2017–2019	25.5 (11.8–34.2)	Live birth rate after LIT RIF: 25/75 (33%) Total: 55/129 (43%)

RIF: recurrent implantation failure; LIT: lymphocyte immunotherapy; n.a.: not applicable; ART: assisted reproductive technology; SD: standard deviation; n.s.: not specified; CI: confidence interval; * mean age 34, standard deviation 3.6 years.

**Table 4:** Summary of selected studies included in the recent meta-analyses (Cochrane and Liu et al.) and data reported by Sarno et al. on live birth rates after LIT in couples with recurrent miscarriage in comparison to our study.

Author	Year	LIT Group *n* (%)	Control *n* (%)	Follow-Up Period (Months)	Comment
Yanping et al.	2011	41/49 (84)	24/45 (53)	*	Intradermal LIT from partner’s blood, before and during pregnancy Control: No treatment
Lin et al.	2012	33/42 (79)	17/42 (41)	*	Subcutaneous LIT from partner’s blood, before and during pregnancy. Control: No treatment
Aiwu et al.	2013	250/297 (84)	254/591 (43)	*	Subcutaneous LIT from the third party or partner’s blood, before and during pregnancy. Control: No treatment
Sarno et al.	2019	452/752 (60)	114/344 (33)	12	Intradermal LIT from partner’s blood, before and/or during pregnancy. Control: No treatment
Günther et al.	2021	RM: 17/32 (53) Total: 55/129 (43)	none	12	Intradermal LIT from partner’s blood, before pregnancy.

LIT: lymphocyte immunotherapy; RM: recurrent miscarriage * No available information about the follow-up period (paper in Chinese, only abstract in English).

## Data Availability

The datasets analyzed for the current study are available from the corresponding author on reasonable request.

## Abbreviations

RIF	Recurrent implantation failure
RM	Recurrent miscarriage
ASRM	American Society for Reproductive Medicine
ART	Assisted reproductive technology
LIT	Lymphocyte immunotherapy
HLA	Human leukocyte antigen
HPA	Human platelet antigen
FNAIT	Fetal and neonatal alloimmune thrombocytopenia
CMV	Cytomegalovirus
uNK cells	Uterine natural killer cells
VEGF	Vascular endothelial growth factor
INF-gamma	Interferon gamma
Ab2	Anti-idiotypic antibodies
MLR-Bf	Mixed lymphocyte reaction blocking antibodies
Treg	Regulatory T cells
